# Gene-Expression Patterns of Tumor and Peritumor Tissues of Smoking and Non-Smoking HPV-Negative Patients with Head and Neck Squamous Cell Carcinoma

**DOI:** 10.3390/biomedicines12030696

**Published:** 2024-03-21

**Authors:** Anna Soboleva, Irina Arutyunyan, Enar Jumaniyazova, Polina Vishnyakova, Daria Zarubina, Eldar Nimatov, Andrey Elchaninov, Timur Fatkhudinov

**Affiliations:** 1 Research Institute of Molecular and Cellular Medicine, Patrice Lumumba Peoples’ Friendship University of Russia (RUDN), 117198 Moscow, Russia; 2 Avtsyn Research Institute of Human Morphology of Federal State Budgetary Scientific Institution “Petrovsky National Research Centre of Surgery”, 117418 Moscow, Russia; 3 Federal State Budget Institution, National Medical Research Center for Obstetrics, Gynecology and Perinatology Named after Academician V.I. Kulakov of Ministry of Healthcare of the Russian Federation, 117513 Moscow, Russia; 4 P. Hertsen Moscow Oncology Research Institute, National Medical Research Radiological Centre of the Ministry of Health of the Russian Federation, 125284 Moscow, Russia

**Keywords:** head and neck squamous cell carcinoma, tobacco smoking, tumor, peritumor tissue, gene expression

## Abstract

We studied the gene-expression patterns in specimens of tumor and peritumor tissue biopsies of 26 patients with head and neck carcinomas depending on smoking status. Histological and immunohistochemical examinations verified that all tumors belonged to the “classical” subgroup of head and neck carcinomas, and the HPV-negative tumor status was confirmed. The expression of 28 tumor-associated genes determined by RT-PCR was independent of patients’ sex or age, TNM status, degree of differentiation, or tissue localization. Moreover, in peritumor tissue, none of the 28 genes were differentially expressed between the groups of smoking and nonsmoking patients. During oncotransformation in both studied groups, there were similar processes typical for HNSCC progression: the expression levels of paired keratins 4 and 13 were reduced, while the expression levels of keratin 17 and *CD44* were significantly increased. However, further investigation revealed some distinctive features: the expression of the genes *EGFR* and *TP63* increased significantly only in the nonsmoking group, and the expression of *IL6*, *CDKN2A*, *EGF*, and *PITX1* genes changed only in the smoking group. In addition, correlation analysis identified several clusters within which genes displayed correlations in their expression levels. The largest group included 10 genes: *TIMP1*, *TIMP2*, *WEE1*, *YAP*, *HIF1A*, *PI3KCA*, *UTP14A*, *APIP*, *PTEN*, and *SLC26A6*. The genetic signatures associated with smoking habits that we have found may serve as a prerequisite for the development of diagnostic panels/tests predicting responses to different therapeutic strategies for HNSCC.

## 1. Introduction

Head and neck squamous cell carcinoma (HNSCC) is a heterogeneous group of tumors that ranks sixth in terms of incidence among all malignant neoplasms [1]. Despite improvements in the clinical outcomes of many tumor types, the overall 5-year survival rate in HNSCC does not exceed 40–50% due to the low efficacy of therapy. Researchers’ interest in this cancer’s nosology is due to the increasing number of young patients, resistance of tumors to the available antitumor drugs, and frequent recurrences [2].

HNSCC is traditionally divided into two subtypes: HPV-positive (HPV+) and HPV-negative (HPV−). HPV+ and HPV- HNSCCs are now largely considered as two distinct cancers that have distinct clinical and molecular biology despite their histological similarities [3,4]. Compared to the HPV-positive subtype, HPV-negative tumors are characterized by a higher mutational load and chromosomal aberrations with different profiles with respect to copy number variation [3,4,5].

The excessive consumption of alcohol or tobacco products and the exposure to environmental pollutants are primary factors in the development of HPV- HNSCC [5,6]. Tobacco, particularly tobacco smoke, is rich in polycyclic aromatic hydrocarbons and nitrosamines, which are known human carcinogens [5,7]. These toxic chemicals are associated with a strongly increased risk of HNSCC [5,7], negatively affect the pharmacodynamics of anticancer drugs [8,9,10], and reduce the efficacy of radiation [11,12] and immune therapy [13,14]. Tobacco smoking, both before and after cancer diagnosis, is an established negative prognostic factor for patients with HNSCC [4,14].

The high diversity and heterogeneity of HNSCC make it necessary to create an expanded classification of HNSCC to identify the key targets to develop effective cancer therapeutics and treatment strategies. For example, retrospective studies have been able to stratify patients with oropharyngeal cancer based on HPV status, tumor stage, nodal stage, and smoking history into risk groups with differing risks of death or distant disease. The authors of the study justify the necessity to select individualized therapy for patients, taking smoking status into account as one of the key factors, because selected patients, namely nonsmokers with a less advanced nodal stage, may be over-treated with current treatment paradigms, and the de-intensification of curative therapy is a current research focus for these patients [4,15].

Studies investigating the effect of tobacco smoke on cancer cell lines in vitro have shown the induction of a more malignant tumor phenotype by increasing the proliferation, migration, invasion, and angiogenesis by activating prosurvival cellular pathways [16]. However, our knowledge of the distinctive features of the transcriptomic profile of normal and tumor tissues of patients depending on tobacco-use status remains incomplete. The present study presents an evaluation of the expression profile of 28 cancer-related genes in tumor and peritumor tissue from smoking and nonsmoking HPV-negative HNSCC patients.

## 2. Materials and Methods

### 2.1. Patient Characterization and Ethical Approval

The biopsy material of tumor and peritumor tissue (located 1 cm from the tumor border) of patients was obtained from P. Hertsen Moscow Oncology Research Institute. All study participants were provided with patient-adapted information, and all patients signed informed consent to participate in the study. The study protocol, patient information, and consent form were approved by an independent ethics committee before patients were included in the study (Extract from Minute No. 634 of the Ethics Committee Meeting of 17 November 2021 and Extract of Minute No. 684 of the Ethics Committee Meeting of 2 March 2022). The study complies with the ethical standards developed in accordance with the World Medical Association Declaration of Helsinki “Ethical Principles for Scientific Medical Research Involving Human Subjects”, as amended in 2000, and the “Rules of Clinical Practice”. Participants were identified by patient number only.

Eligibility criteria were as follows. Inclusion criteria: (1) 18 years and older; (2) diagnosis of primary squamous cell carcinoma of the head and neck. Exclusion criteria: (1) HPV+ status; (2) insufficient biopsy volume for examination; (3) history of chemotherapy or radiotherapy; (4) ongoing or active infection; (5) time for transporting biopsy specimens to the laboratory being more than 4 h.

Biomaterial from 26 patients obtained during the surgical stage of treatment was used in the study; information about the patients is presented in Table 1. All patients were diagnosed and histologically verified as having head and neck squamous cell carcinoma, of the HPV-negative subtype, without distant metastases. The nonsmoking patient group consisted of fifteen never-smoking patients (never smoked or smoked less than 100 cigarettes in their lifetime) and two patients who had stopped smoking more than 11 years prior to diagnosis [17]. All smoking patients consisted of current smokers when diagnosed.

Here, we must highlight the limitations of the study. The proportion of HNSCC patients who smoke is usually higher than that of nonsmokers, and the proportion of HNSCC patients with lymph node metastases is usually up to 40–50%. However, our prospective study does not reflect these statistics, as the enrollment of patients is not a random selection due to the strict exclusion criteria.

Biopsies of tumor and peritumoral tissues were placed in a Custodiol HTK preservation solution and transported to the research laboratory at a temperature of 2–4 °C for no more than 4 h. Next, every bioptate was divided into fragments: (1) for DNA isolation and HPV status testing, (2) for RNA isolation and gene expression profile analysis, (3) and for cryosectioning and histological/IHC examination.

### 2.2. Testing the HPV Status of the Patients

DNA was isolated from the patients’ biological material using “ExtractDNA Blood and Cells” reagent kit (Eurogen, Moscow, Russia). The HPV status of patients was tested using the reagent kit “HPV quant-21”, designed for the detection, typing, and quantification of human papillomavirus DNA of low (HPV 6, 11, 44) and high (HPV 16, 18, 26, 31,33, 35, 39, 45, 51, 52, 53, 56, 58, 59, 66, 68, 73, 82) carcinogenic risk (DNA-Technology, Moscow, Russia).

### 2.3. Histopathologic and Immunohistochemistry Analysis

For cryosectioning, tissues were embedded in OCT-medium (Tissue-Tek, Sakura, Torrance, CA, USA) and frozen at −80 °C. Cryosections that were 7 μm thick were sliced in a Leica CM 1900 cryostat (Leica, Frankfurt, Germany), collected on SuperFrost-plus microscope slides (Menzel, Braunschweig, Germany), dried at room temperature for an hour, and stored refrigerated until use.

For routine histologic examination, the cryosections were stained with hematoxylin and eosin (BioVitrum, Saint-Petersburg, Russia).

For IHC examination, the cryosections were stained with antibodies to E-cadherin (ab1416, Abcam, Cambridge, UK), vimentin (ab8978, Abcam), CD44 surface receptor (DF6392, Affinity, London, UK), keratin 13 (PAB875Hu01, Cloud Clone, Wuhan, China), and keratin 17 (ab53707, Abcam). Cell nuclei were counterstained with DAPI (Sigma, Darmstadt, Germany). The fluorescence was examined with a Leica DM 4000B fluorescence microscope and LAS AF v.3.1.0 build 8587 software (Leica Microsystems, Wetzlar, Germany).

### 2.4. Determination of Gene Expression Levels

A list of 28 cancer-related genes was compiled by analyzing the publicly available databases The Cancer Genome Atlas Program (TCGA), International Cancer Genome Consortium (ICGC), and Clinical Proteomic Tumor Analysis Consortium (CPTAC). In these databases, patients with head and neck squamous cell carcinoma (without division into smokers and nonsmokers) with a poor prognosis were selected. The most differentially expressed genes with decreased (*SLC13A4*, *PITX1*, *KIAA1045*, *STX19*, *UBE2Z*, *FCGBP*, *TMPRSS11D*, *KRT13*, *PRSS27*, *EPS8L1*, *FAM57A*, *C4orf23*, *ZNF395*, *ABCA12*) and increased *(RNF5P1*, *APIP*, *SLC26A6*, *SPESP1*, *RNF121*, *ARIH2*, *C3orf35*, *DDX31*, *UBE2A*, *CANT1*, *HIST1H4E*, *DNASE1L1*, *TMX4*, and *UTP14A*) expression were identified. Each of them was then tested, and a correlation analysis was carried out. The genes *APIP*, *SLC26A6*, and *UTP14A* showed at least some significant expression among the upregulated genes, and *EPS8L1*, *KRT13*, and *PITX1* showed some significant expression among the downregulated genes. All other genes mentioned (Table 2) were taken from published literature sources [2,3,5,6,18,19,20].

Gene transcription rates were assessed by quantitative real-time reverse transcription PCR. Total RNA was isolated from samples frozen in RNAlate using the RNeasy Plus Mini Kit (QIAGEN, Hilden, Germany). The calculated concentration of purified RNA in the eluate was 0.1 g/L; RNA integrity was confirmed by electrophoresis in a 1% agarose gel stained with ethidium bromide. The synthesis of cDNA from total RNA matrix was performed using the off-the-shelf MMLV RT kit (Eurogen). The resulting cDNA was subjected to PCR in triplicate using the qPCRmix-HS SYBR reagent kit containing SYBR Green I fluorescent intercalating dye (Eurogen). PCR primers were designed using the online resource Primer-BLAST according to generally accepted requirements. The primers (Table 2) were synthesized by Eurogen.

Gene expression levels were quantified using the threshold cycle (Ct) method: characteristic values were automatically generated by nonlinear regression analysis, and the relative expression values were calculated using the approach originally introduced by Pfaffl [21] using *GAPDH* as the reference target as it had exhibited high expression stability.

### 2.5. Statistical Analysis

The data were analyzed using GraphPad Prism v. 8.4.3 (GraphPad Software, Inc., San Diego, CA, USA) and StatTech v. 2.8.8 (Stattech, Kazan, Russia). To compare relative gene expression, ANOVA on the Ranks rank analysis of variance was used, and posthoc pairwise comparisons were performed using Dunn’s test. The strength and direction of association between two ranked variables were evaluated using the Spearman rank correlation coefficient. The differences were significant at a 5% significance level. Data were presented as median and interquartile range.

## 3. Results

### 3.1. Verification of Patients’ HPV Status

The study confirmed the HPV-negative status of all examined specimens.

### 3.2. Histological Examination of Tumor and Peritumor Tissues

**Figure 1:**
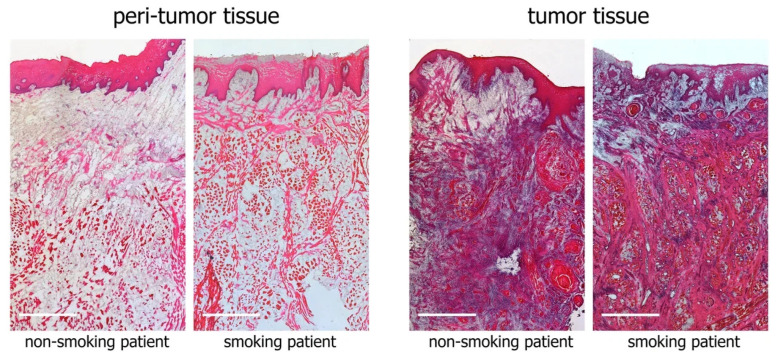
Tumor and peritumor tissues from a smoking and a nonsmoking patient with tongue cancer. Hematoxylin and eosin staining; scale bar represents 1 mm.

Histological examination confirmed the development of squamous cell cancer in tumor tissue bioptates and showed the absence of tumor cell invasion in biopsy specimens of peritumor tissue. As an example, Figure 1 shows microphotographs of tumor and peritumor tissues from a smoker and a nonsmoker with tongue cancer. We found no morphologic differences in the bioptates between smoking and nonsmoking patients with similar tumor localization and TNM status.

**Figure 2:**
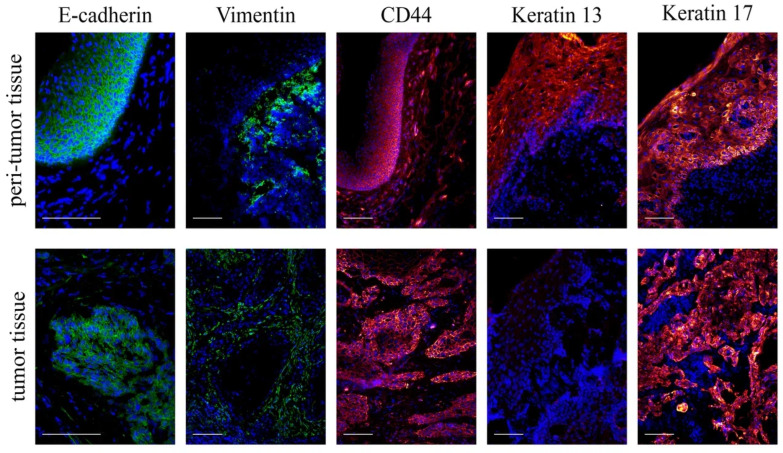
Tumor and peritumor tissues from patients with laryngeal cancer. The cryosections were stained with antibodies for E-cadherin, vimentin, CD44, keratin 13, and keratin 17. Cell nuclei were counterstained with DAPI. Fluorescence microscopy; scale bar represents 100 μm.

An immunohistochemical study in all cases confirmed the epithelial origin of neoplasms: cells expressing vimentin (a marker of cells of mesenchymal origin) were located only in connective tissue layers surrounding clusters of invasive cells expressing epithelial cell adhesion protein E-cadherin. In the peritumor tissue, E-cadherin was expressed only by the cells of mucosal epithelium covering the underlying tissues, where vimentin+ cells were located (Figure 2).

Cancer cells were stained intensely with antibodies for CD44, as were normal epithelial cells in the peritumor tissue; however, less intense but clearly visible staining was also characteristic of the stromal cells of the organ mucosa (Figure 2).

Normal peritumor epithelial cells expressed keratins 13 and 17, whereas in tumor tissue, the surface and invading epithelial cells retained the KRT17+ phenotype, but KRT13 expression disappeared (Figure 2).

### 3.3. Gene Expression Analysis

We found no statistically significant differences in the expression of 28 genes depending on the sex or age of the patients, TNM status, degree of differentiation, or tissue localization.

**Figure 3:**
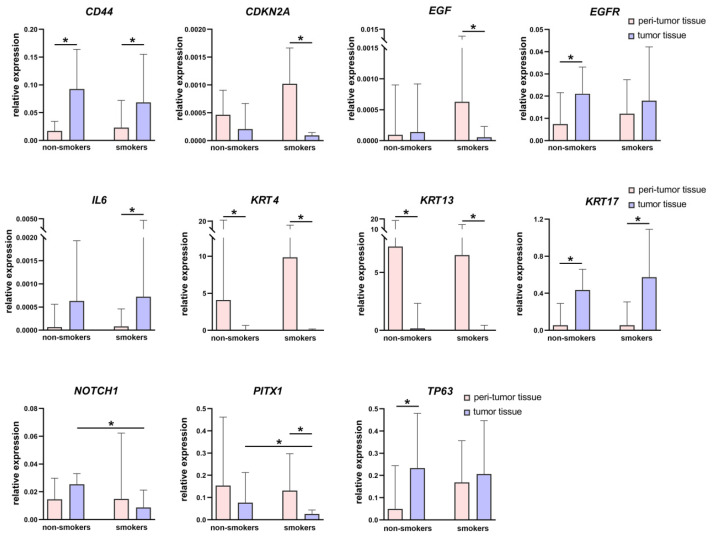
Relative expression levels of the 11 differently expressed tumor-associated genes in tumor and peritumor tissues of patients with HNSCC according to their smoking status. Data are presented as median and interquartile range. * *p* < 0.05.

**Figure 4:**
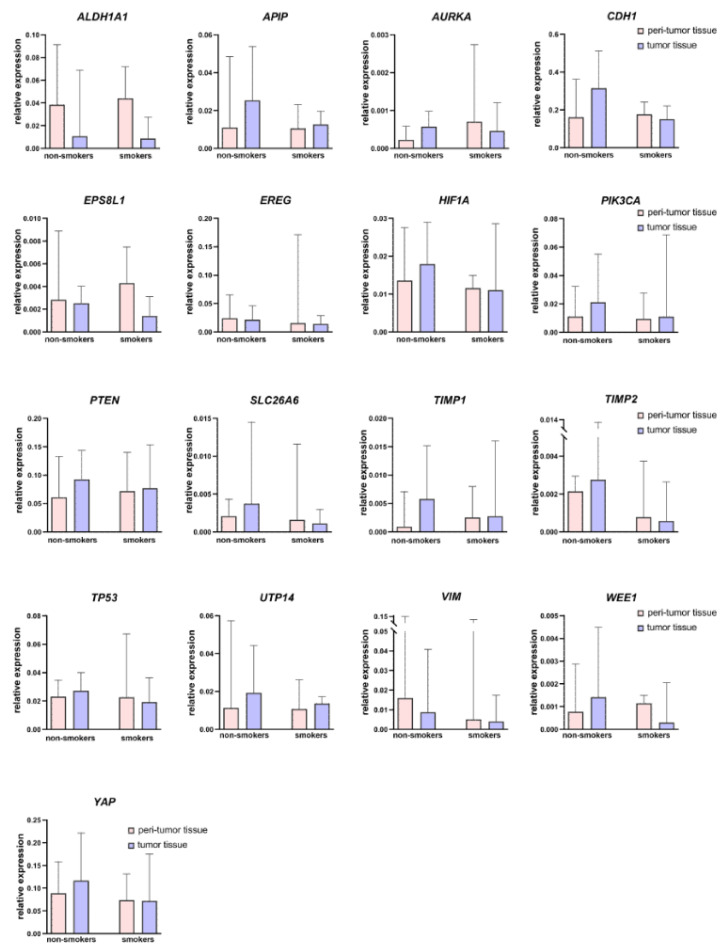
Relative expression levels of the 17 tumor-associated genes in tumor and peritumor tissues of patients with HNSCC according to their smoking status. Data are presented as median and interquartile range. No statistically significant differences were identified.

Figure 3 and Figure 4 present data on the relative expression levels of selected tumor-associated genes in peritumor and tumor tissue of smoking and nonsmoking patients with HNSCC.

Interestingly, in peritumor tissue, none of the 28 genes were differentially expressed between the groups of smoking and nonsmoking patients.

A comparison of tumor and peritumor tissues showed that during oncotransformation in both studied groups, there were similar processes: the expression levels of keratins 4 and 13 were reduced, while the expression levels of keratin 17 and *CD44* were significantly increased. At the same time, some notable differences were found. Thus, only in the group of nonsmoking patients did we find an increased expression level of *TP63* and *EGFR* genes in tumor tissue compared to peritumor tissue. *IL6* level increased in the tumor tissue of smoking patients (in the group of nonsmoking patients, we observed a similar trend, but the changes did not reach statistically significant values), and the expression levels of *EGF*, *CDKN2A*, and *PITX1* were decreased. In addition, the expression level of *NOTCH1* and *PITX1* was lower in the tumor tissue of smoking patients compared to the tumor tissue of nonsmoking patients.

**Figure 5:**
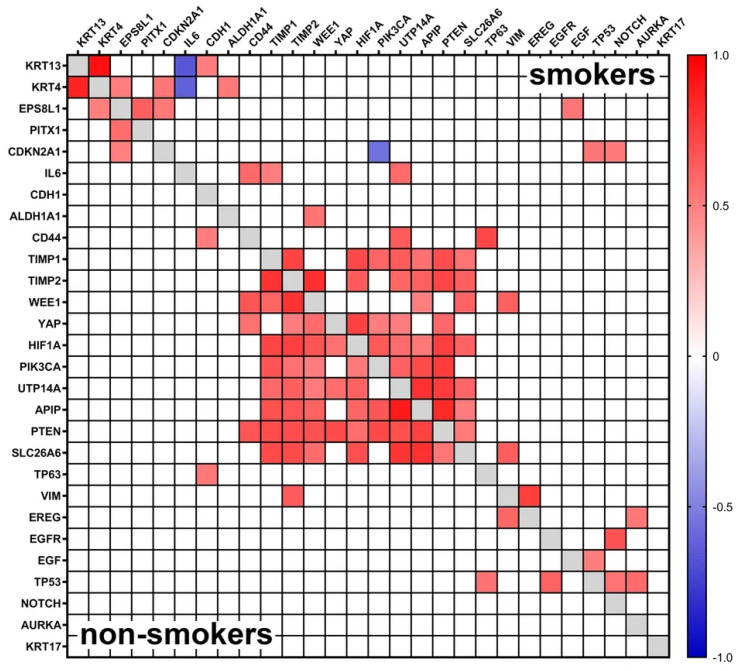
A heat map depicting the strength of gene expression correlation in tumor and peritumor tissues of smoking and nonsmoking patients. The map represents the points for which a statistically reliable “significant”, “strong”, or “very strong” (according to the Chaddock assessment scale) density of the correlation was found.

Figure 5 shows a heat map depicting the strength of gene expression correlation in tumor and peritumor tissues of smoking and nonsmoking patients. The map represents the points for which a statistically reliable “significant”, “strong”, or “very strong” density (according to the Chaddock assessment scale) of the correlation was found.

We were able to detect several groups of interconnected genes. Regardless of the smoking status of patients, we found a very strong direct association for keratins 4 and 13 (*p* < 0.001); the same group included the genes *EPSF8L1*, *PITX1*, and *CDKN2A*. In smoking patients, individual genes in this group were additionally found to be associated with *IL6*, *CDH1*, *ALDH1A1* (via keratins 4 or 13), *TP53*, *NOTCH1*, and *EGF* (via *EPSF8L1* or *CDKN2A*) genes.

A large cluster of 10 genes (*APIP*, *HIF1A*, *PIK3CA*, *PTEN*, *SLC26A6*, *TIMP1*, *TIMP2*, *UTP14*, *WEE1*, and *YAP*) was identified, with multiple significant or strong positive correlations within the group, indicating that these genes are included in cognate signaling pathways.

Interestingly, no correlation was found between the *KRT17* gene and any of the other 27 genes.

## 4. Discussion

It is well known that due to the complex structure of the head and neck organs, tumors of epithelial, mesenchymal, lymphoid, and melanocytic origin can occur in this anatomical region, but the most frequent (more than 90% of cases) malignant neoplasm in this region is squamous cell cancer [22]. HNSCC develops from the mucosal epithelium, passing through the stages of hyperplasia, dysplasia, in situ carcinoma, and invasive carcinoma. The peculiarities of HNSCC progression usually lead to late diagnosis, when the disease is detected at the stage of invasive carcinoma [6]. In this case, the histopathological examination of biopsy specimens becomes the main prognostic tool capable of providing important information about the tumor tissue architectonics to correct further treatment tactics of the patient [23]. The absence of vimentin-expressing cells in the foci of invasion confirmed that all the tumors studied belonged to the so-called “classical” rather than “mesenchymal” subgroup of HNSCC [2].

One of the diagnostic markers of HNSCC development is a change in the expression profile of keratins, proteins of intermediate filaments of epithelial cells: as oncotransformation progresses in epithelial cells, the expression of *KRT4* and *KRT13* decreases, and the expression of *KRT17* increases. The keratin intermediate filaments are assembled from heterodimers formed by two types of keratins, I and II. The distinction between type I (acidic or subfamily A) keratins and type II (basic or subfamily B) keratins is based on the pH at which the proteins are neutral. The basic keratin 4 forms heterodimers with the acidic keratin 13 [24], which explains the very strong correlation between the expression levels of *KRT4* and *KRT13* genes that we found. In normal mucosa, *KRT4* and *KRT13* are expressed in the suprabasal layers of multilayer epithelium. In the development of oral squamous cell carcinoma [18,25] and squamous cell carcinoma of mobile tongues [26], a decrease in the expression of *KRT4* and *KRT13* has been shown. The degree of decrease in expression correlates with the degree of oncotransformation of the epithelium [18,25] and therefore is associated with poor prognosis [26]. The appearance of *KRT17* is typical for HNSCC and is often used to verify the diagnosis [18]. For example, an increased expression level of *KRT17* was shown for 101 out of 105 (96.2%) oral squamous cell carcinoma specimens examined, and the expression level was higher in well-differentiated oral SCC compared to moderately/poorly differentiated oral SCC [27]. In our study, we confirmed a significant increase in *KRT17* expression and a crucial decrease in *KRT13* and *KRT4* expression in tumor tissues relative to the peritumor region in all patients studied, regardless of tumor grade or patient smoking status. Furthermore, IHC examination of the tissue showed that *KRT13* expression indeed almost completely disappears in mucosal epithelial cells in tumor tissue biopsy specimens, and all cancer cells have a KRT17+ phenotype. *KRT17* is expressed at a high level by cells of HNSCC tumor lines and is absent in normal epithelium but is induced when it is damaged at the stage of hyperproliferation [28]. Microscopic sections of peritumor tissue show that the mucosal epithelium expresses *KRT17*, which may indicate damage or the initial stage of oncotransformation of cells located near the tumor site.

We also evaluated the expression level of vimentin, another intermediate filament protein widely expressed in cells of mesenchymal origin [29,30]. Modulations of cytoskeletal organization correlate to tumorigenesis, invasive ability, and epithelial–mesenchymal transition (EMT), the latter process accompanied by the loss of epithelial markers and the gain of vimentin [29,30,31]. The second hallmark EMT protein is E-Cadherin, an adhesion molecule that functions to maintain cell–cell contacts. During EMT, E-cadherin function is reduced through downregulation or delocalization from the cell membrane, epithelial cells lose polarity, and that is an early stage of EMT [32,33]. Increased vimentin and decreased E-cadherin expression in oral cancers are associated with metastasis and disease progression [31]. For example, in a study including 26 patients with stage II-IV HNSCC, it was shown that 100% of primary tumors with the low E-cadherin and high Vimentin signature developed distant metastases compared to a 44% metastasis rate for primary HNSCC tumors with an incomplete or null EMT signature [33]. In our study, 100% of samples were obtained from patients with M0 status, which explains the absence of significant changes in the expression levels of *VIM* and *CDH1* genes (this gene encodes E-Cadherin protein). Moreover, the preserved expression level of *CDH1* is a positive prognostic factor indicating a low probability of recurrence in patients with HNSCC [34,35].

Interestingly, *VIM* expression positively correlated with the *EREG* gene (encodes epiregulin protein) in both groups of patients, and also with the *AURKA* gene in smokers. Although all three of these genes were not significantly altered in tumor and peritumor tissues, their association was shown by us for HNSCC for the first time. *EREG* is also known to be involved in EMT activation and the progression of salivary adenoid cystic carcinoma [19] and oral squamous cell carcinoma [36].

The phenomenon of EMT is an intricate process with a timely interplay of a variety of complex network-comprising inducers, for example, transcription factor *HIF1A* or intracellular signal transducer phosphoinositide 3-kinase [30]. Along with other signs of EMT absence, we also found no significant changes in the expression levels of *HIF1A* and *PIK3CA* in the studied samples.

The surface receptor of hyaluronic acid and matrix metalloproteinases CD44 is involved in the processes of cell migration; a high level of *CD44* expression in head and neck tumors is associated with the risk of metastasis and poor prognosis [6,37]. The integral membrane glycoprotein CD44 is considered one of the cancer stem cell (CSC) markers for HNSCC [22,37] and is widely used to assess the relevance of organoids [38] or immortalized cell lines derived from these tumors [39]. Another putative marker of CSCs is the aldehyde dehydrogenase ADLH1A1. Increased *ALDH1A1* expression in HNSCC correlates with a low degree of tumor cell differentiation, metastasis to lymph nodes, and duration of a recurrence-free period [40], which allowed us to propose *ALDH1A1* as a prognostic biomarker for oropharyngeal squamous cell carcinoma [41]. RT-PCR did not reveal any change in *ALDH1A1* expression level but confirmed an increased level of *CD44* expression in tumor tissue of smokers and nonsmokers, while IHC study demonstrated that *CD44* is expressed not only by normal and tumor epithelial cells but also by cells of the organ stroma, which casts doubt on the possibility of using this receptor to mark specific populations of tumor cells.

In biopsy specimens of smoking patients, we found a significant association of *CD44*, *KRT4*, and *KRT13* expression with *IL6* gene expression, the level of which was increased in tumor tissue of smokers compared to peritumor tissue. This pro-inflammatory cytokine plays an important role in a number of cellular processes, including proliferation, survival, differentiation, migration, and invasion [42]. High serum IL-6 levels are associated with worse tolerance to chemotherapy in some types of cancer, as well as a poor prognosis in patients with HNSCC [5,43,44]. In tumors, IL6 is involved in the recruitment of mesenchymal and endothelial cells, contributing to tumor progression [45]. IL6 can be produced by various cell populations of the cancer microenvironment. It can easily leak out and affect distant tissues and cells, thus contributing to the formation of a premetastatic niche; therefore, IL-6 seems to be a promising target in antitumor therapy [42]. At the same time, our results show that a higher efficacy of anti-IL-6 therapy can be expected in a cohort of smoking patients, where this cytokine plays an important role and interacts with a number of other tumor-associated genes.

In both patient groups, we found an association between the expression levels of *KRT4* and EGFR pathway substrate 8 (EPS8) signaling adaptor L1 (*EPS8L1*). We observed a trend toward decreased expression levels of *EPS8L1* in smokers. The exact function of this protein is unknown, but not so long ago, there appeared data on its high methylation level in smoking patients with chronic obstructive pulmonary disease [46], as well as on suppression of *EPS8L1* in radioresistant clones of pancreatic cancer cell lines [47]. In biopsy specimens of both groups, we found correlations of *EPS8L1* expression with *PITX1* and *CDKN2A*, and in smokers also with *EGF*. These thee genes were suppressed in the tumor tissue of smokers compared to peritumor tissue.

Paired-like homeodomain transcription factor 1 (PITX1) belongs to the highly conserved homeobox genes that play a critical role in establishing cell identity during the spatial and temporal dimensions of animal growth and development, but not so long ago, much evidence has been found for the involvement of PITX1 in cancer development. It is assumed that it functions as a tumor-suppressor gene in several human cancer types: decreased expression level of *PITX1* is observed in various malignant tumors, e.g., HNSCC [48,49], gastric cancer [50], lung cancer [51], and colorectal carcinoma [52]. However, there is contrary evidence that *PITX1* expression is upregulated in breast cancer [53,54], prostate cancer [55], and lung cancer [56]. Thus, *PITX1* expression is tumor-type-specific. For patients with HNSCC, low levels of *PITX1* are a possible predictive biomarker of chemosensitivity: immunohistochemical analysis performed to examine the expression levels of *PITX1* in 47 cases of HNSCC showed that the lowest level of *PITX1* production was observed in the group of patients with stable or progressive disease, and the highest in the complete response group [48]. In our study, we showed for the first time that the significant suppression of *PITX1* is characteristic of smoking patients with HNSCC, and the level of *PITX1* expression in the tumor tissue of smokers was lower than in nonsmoking patients.

Cyclin-dependent kinase inhibitor 2A (CDKN2A) encodes proteins that are critical in cell cycle regulation. Some types of cancers, such as HNSCC, neural system tumors, gastro-intestinal cancer, breast cancer, and lung adenocarcinoma, are reported in association with *CDKN2A* mutations [57]. *CDKN2A* mutations leading to loss of function are significant predictors of mortality among patients with recurrent or metastatic HNSCC receiving immunotherapy [58]. Our data on the decreased expression of *CDKN2A* in tumors of smokers indicate the necessity of taking into account the smoking status of patients with HNSCC when prescribing immunotherapy.

In the group of smokers, we found a direct correlation within the *CDKN2A*, *TP53*, and *NOTCH1* gene groups. *NOTCH1*, one of the most frequently mutated genes in HNSCC, encodes a transmembrane receptor that plays an important role in cell and tissue development. The structural characterization of *NOTCH1* mutations in HNSCC demonstrates that most are predicted to cause loss of function, in agreement with *NOTCH1*’s role as a tumor suppressor in this cancer. This loss of NOTCH1 signaling can drive HNSCC tumorigenesis and clinical aggressiveness [59]. In our study, the level of *NOTCH1* expression in tumor tissue of smokers was lower than in the group of nonsmoking patients, but this observation is difficult to interpret, since the data on the use of *NOTCH1* as a predictor of HNSCC outcome are contradictory [59].

In the group of smokers, we were surprised to observe a decrease in *EGF* (epidermal growth factor) gene expression, whereas in nonsmokers, the expression of its receptor *EGFR* was expectedly increased. *EGFR* is overexpressed in more than 90% of HNSCC cases, and its increased expression correlates with poor outcome [60]. Whole-genome profiling of a large number of HNSCC tumors has identified focal *EGFR* amplification as canonical genomic abnormalities in the development of HNSCC [61]. Selective *EGFR* inhibitors have been approved by the FDA as a therapy for HNSCC [62,63]. At the same time, the difficulty in investigating the *EGFR* signaling pathway is due to the fact that the ligands for EGFR include at least five molecules, i.e., AREG (amphiregulin), EREG (epiregulin), EGF, heparin-binding EGF-like growth factor, and beta-cellulin [19,64]. The observed decrease in *EGF* levels in smokers may be explained by a shift in the balance of ligands, but this assumption requires further investigation.

In addition to *EGFR*, the level of *TP63*, a master regulator of epithelial biology, proliferation, and differentiation, was significantly increased in the tumors of nonsmoking patients. It is overexpressed in the majority of HNSCC cases: p63+ cells are present in 96–100% of squamous cell carcinoma cases regardless of its origin [65]. Our data on the positive correlation between *TP63* and *CD44* are supported by the results of other researchers, who suggest that the *TP63-CD44* pathway is a negative prognostic factor of HNSCC patient survival [66].

The most interesting observation, in our opinion, is the detection of a cluster of 10 genes within which the genes were interconnected by direct significant or strong correlation. This cluster was found in peritumor and tumor biopsy specimens from both smoking and nonsmoking patients, suggesting its significance in the development of HNSCC. The group included *TIMP1*, *TIMP2*, *WEE1*, *YAP*, *HIF1A*, *PI3KCA*, *UTP14A*, *APIP*, *PTEN*, and *SLC26A6* genes.

Tissue inhibitors of metalloproteinases (TIMPs) affect ECM remodeling, apoptosis, cancer cell growth, and immune surveillance, which in turn can promote invasion and metastasis, so TIMPs exert an important role in tumor pathogenesis and patient survival in various cancers, including head and neck cancers [67].

WEE1 is a protein kinase that regulates the G2 checkpoint and prevents entry into mitosis in response to DNA damage and is frequently overexpressed in various tumors, including laryngeal squamous cell carcinoma [68]. The increased expression of *WEE1* in most cases is associated with HPV+ status of a tumor cell line or a patient with HNSCC [69]; similarly, HPV infection is also associated with increased frequency of *PIK3CA* and *PTEN* gene mutations [70], which explains the absence of changes in the expression level of these genes in our study.

Downstream targets of YAP (Yes-associated protein 1) are determined by its interactions with multiple transcriptional and epigenetic regulators whose expression and activity are dynamically modulated by oncogenic signaling. YAP acts as a transcription cofactor regulating the expression of genes involved in cell proliferation, EMT, and cell migration, all of which contribute to the pro-tumorigenic phenotype [20,71,72]. At the same time, it was shown that in different oral SSC cell lines, *YAP* can be overexpressed or, on the contrary, reduced due to phosphorylation and translocation from the cell nucleus to the cytoplasm [73]. *YAP*-regulated transcriptional signatures specific to each stage of HNSCC development and progression in vivo are not well known [20].

UTP14A (U three protein 14A) plays a key role in the synthesis of ribosomes and 18S rRNA; however, in the last few years, data have appeared on the association of this gene with some types of tumors—hepatocellular carcinoma, colorectal carcinoma, and esophageal squamous cell carcinoma [74]. The exact mechanisms of its activity are unknown, but we have shown for the first time that the expression of this gene correlates with genes involved in the development of HNSCC.

Apaf-1 interacting protein (APIP) functions as an endogenous inhibitor of apoptotic cell death by inhibiting caspase activity. Its upregulation leads to chemotherapeutic resistance in cancer cells [75]. In our work, we did not find changes in *APIP* level in HNSCC patients, but it can be assumed that evaluation of the expression level of this gene will be of interest to researchers when studying the mechanisms of tumor therapeutic resistance.

Another non-obvious gene that fell into this cluster was *SLC26A6*. The solute-linked carrier 26 (SLC26) protein family comprises multifunctional transporters of substrates that include oxalate, sulfate, and chloride and play important roles in renal physiology and pathophysiology [76]. One member of this family, SLC26A6, is of particular interest because it has some non-canonical properties. For example, it was recently shown that SLC26A6 acts as an oncogene in hepatocellular carcinoma [77] and lung cancer [78]. The role of SLC26A6 in HNSCC is completely unexplored.

## 5. Conclusions

Thus, we have shown that smoking or the absence of this harmful habit leads to some differences in the expression profile of several tumor-associated genes in patients with head and neck cancer. Although none of the 28 genes was differentially expressed between the groups of smoking and nonsmoking patients in peritumor tissue, the expression level of *NOTCH1* and *PITX1* was significantly lower in the tumor tissue of smokers compared to that of nonsmokers. In addition, several clusters within which genes displayed correlation in their expression levels were identified. These clusters included both well-studied genes and genes whose role in HNSCC tumorigenesis is very poorly understood. Currently, there are not many antitumor drug treatment options for HNSCC. The completion of the molecular genetic profile of this nosology will allow us to find targets for new therapeutic agents. Moreover, the genetic signatures associated with smoking habits that we have found may serve as a prerequisite for the development of diagnostic panels/tests predicting the response to different therapeutic strategies.

## Figures and Tables

**Table 1:** Characteristics of patients.

Characteristics	Nonsmokers, *n* (%)	Smokers, *n* (%)	Total, *n* (%)
*Smoking status*	17 (65.3%)	9 (34.7%)	26 (100.0%)
*Sex*			
Males	8 (47.1%)	7 (77.8%)	15 (57.7%)
Females	9 (52.9%)	2 (22.2%)	11 (42.3%)
*Age (in years)*			
Median	59	63	60,5
Range	19–82	50–64	19–82
*T stage*			
cT1	1 (5.9%)	1 (11.1%)	2 (7.7%)
cT2	7 (41.2%)	1 (11.1%)	8 (30.8%)
cT3	4 (23.5%)	2 (22.2%)	6 (23.1%)
cT4	5 (29.4%)	5 (55.6%)	10 (38.4%)
*N stage*			
cN0	16 (94.1%)	7 (77.8%)	23(88.5%)
cN1	0 (0%)	1 (11.1%)	1 (3.8%)
cN2	1 (5.9%)	1 (11.1%)	2 (7.7%)
*Differentiation*			
Well	11 (64.7%)	6 (66.7%)	17 (65.4%)
Moderate	5 (29.4%)	2 (22.2%)	7 (26.9%)
Poor	1 (5.9%)	1 (11.1%)	2 (7.7%)
*Keratinization*			
Keratinizing	11 (64.7%)	7 (77.8%)	18 (69.2%)
Non-keratinizing	6 (35.3%)	2 (22.2%)	8 (30.8%)
*Location*			
alveolar ridge	2 (11.8%)	2 (22.2%)	4 (15.4%)
larynx	2 (11.8%)	3 (33.3%)	5 (19.2%)
oral cavity	6 (35.3%)	1 (11.1%)	7 (26.9%)
mobile tongue	7 (41.1%)	3 (33.3%)	10 (38.5%)

**Table 2:** Oligonucleotide primer sequences.

Gene	(5′ to 3′)	(3′ to 5′)
*ALDH1A1*	GATTGGATCCCCGTGGCGTA	GATTGGATCCCCGTGGCGTA
*APIP*	CGCGCAGGACAAGGAGCAT	CTTCGATGGCGAAGGTCCAC
*AURKA*	AGTGGCGGAGCGTCAAGTC	AGTGGCGGAGCGTCAAGTC
*CDH1*	ACTGATGCTGATGCCCCCAA	ACTGATGCTGATGCCCCCAA
*CDKN2A*	CTGCCCAACGCACCGAATAG	CTGCCCAACGCACCGAATAG
*CD44*	AGGAGCAGCACTTCAGGAGG	AGGAGCAGCACTTCAGGAGG
*EGF*	TTCACTGTCTTGACTCTACTCCACC	TTCACTGTCTTGACTCTACTCCACC
*EGFR*	CCCCCTGACTCCGTCCAGTA	CCCCCTGACTCCGTCCAGTA
*EPS8L1*	GGAAGGGAAAGGACAGCGGA	CTCACCCAGGCAGAACGTCA
*EREG*	TGCTCTGCCTGGGTTTCCAT	TGCTCTGCCTGGGTTTCCAT
*HIF1A*	GCCCATTCCGCGTCTGAGT	GCCCATTCCGCGTCTGAGT
*IL-6*	GGTATACCTAGAGTACCTCCA	GGTATACCTAGAGTACCTCCA
*KRT4*	GATCGCCACCTACCGCAAAC	GATCGCCACCTACCGCAAAC
*KRT13*	GGGACTCATCAGCAGCATCG	GGGACTCATCAGCAGCATCG
*KRT17*	AGATTGCCACCTACCGCCG	AGATTGCCACCTACCGCCG
*NOTCH1*	CCCACTCATTCTGGTTGTCG	CCCACTCATTCTGGTTGTCG
*PITX1*	AACCGCTACCCCGACATGAG	CTGCACTAGGCCGCTGAACT
*PIK3CA*	TTCCGGGGGATTGTAGGCTC	TTCCGGGGGATTGTAGGCTC
*PTEN*	CCCAGTCAGAGGCGCTATGT	CCCAGTCAGAGGCGCTATGT
*SLC26A6*	AGACAGCCAGAGATGCTGCC	GTAGGTGACCACGAAGCCGA
*TIMP1*	CCTTCCAGGTGTTTCCCTGTT	CCTTCCAGGTGTTTCCCTGTT
*TIMP2*	GACCCACAAGGAGATTGGGG	GACCCACAAGGAGATTGGGG
*TP53*	GTGCTTTCCACGACGGTGAC	GTGCTTTCCACGACGGTGAC
*TP63*	GTGTTGGAGGGATGAACCGC	GTGTTGGAGGGATGAACCGC
*UTP14*	TCTGGCTTTGAGCCAACAGG	GACCTCTCAGCCAATTTCCGC
*VIM*	TCAATCGGCGGGACAGCAG	TCAATCGGCGGGACAGCAG
*WEE1*	AACAATGGGCCTCGTCTGGA	AACAATGGGCCTCGTCTGGA
*YAP*	CAGCAACTCCAACCAGCAGC	CAGCAACTCCAACCAGCAGC
*GAPDH*	GCACCGTCAAGGCTGAGAAC	TGGTGAAGACGCCAGTGGA

## Data Availability

Earlier data have not been published anywhere.
